# Direct archaeological evidence for Southwestern Amazonia as an early plant domestication and food production centre

**DOI:** 10.1371/journal.pone.0199868

**Published:** 2018-07-25

**Authors:** Jennifer Watling, Myrtle P. Shock, Guilherme Z. Mongeló, Fernando O. Almeida, Thiago Kater, Paulo E. De Oliveira, Eduardo G. Neves

**Affiliations:** 1 Museum of Archaeology and Ethnology, University of São Paulo, São Paulo, Brazil; 2 Anthropology and Archaeology Program, Institute of Social Science, Federal University of Western Pará, Santarém, Pará, Brazil; 3 Department of Archaeology, Federal University of Sergipe, Aracajú, Sergipe, Brazil; 4 Institute of Geosciences, University of São Paulo, São Paulo, Brazil; 5 Department of Botany, The Field Museum of Natural History, Chicago, Illinois, United States of America; New York State Museum, UNITED STATES

## Abstract

Southwestern Amazonia is considered an early centre of plant domestication in the New World, but most of the evidence for this hypothesis comes from genetic data since systematic archaeological fieldwork in the area is recent. This paper provides first-hand archaeobotanical evidence of food production from early and middle Holocene (ca. 9,000–5000 cal. BP) deposits at Teotonio, an open-air site located on a 40 m-high bluff on the south bank of the Madeira river. Such evidence includes the presence of local and exotic domesticates such as manioc (*Manihot esculenta*), squash (*Cucurbita* sp.) and beans (*Phaseolus* sp.), alongside edible fruits such as *pequiá* (*Caryocar* sp.) and guava (*Psidium* sp.) that point to the beginnings of landscape domestication. The results contribute to an ever-growing number of studies that posit southwest Amazonia as an important centre for early crop domestication and experimentation, and which highlight the *longue-durée* of human impacts on tropical forest biodiversity around the world.

## Introduction

Southwestern Amazonia is considered an independent centre of plant domestication in the New World [1–3]. The seasonal forests of the upper Madeira and Guaporé river watersheds were where manioc (*Manihot esculenta*), peanut (*Arachis hypogea*), anato (*Bixa orellana*), the peach palm (*Bactris gasipaes*), rice (*Oryza sp*.) and, more tentatively, one squash (*Cucurbita maxima*) and one chilli species (*Capsicum baccatum)* were initially domesticated [1,3,4]. Most of these plants have a social and economic importance today that transcends the Amazon, but perhaps the most remarkable of them is manioc, a root crop which feeds at least 500 million people throughout the tropics [5].

Until now, this genetic evidence has been interpreted in the absence of direct evidence for past plant cultivation and management practices. Despite pioneering work done in the 1970s and 1980s that demonstrated continuous records of human occupation for open-air sites, fluvial shell mounds and rock shelters located along the upper Madeira river basin in Brazil [6], it is only recently that systematic research has begun in the region. This paper presents for the first time archaeobotanical data from excavations at Teotonio site, where a sequence of human occupation that spans most of the Holocene was recovered. By employing on-site macrobotanical recovery, phytolith analysis of on-site soils, and phytolith and starch grain analyses of lithic residues pertaining to both the Girau and Massangana phases, we bring evidence of some of the resources that were exploited at Teotonio during the early and middle Holocene, and tie this in with one of the earliest sequences of Anthropogenic Dark Earths yet discovered in the Amazon basin.

## Study area

### Upper Madeira

**Fig 1:**
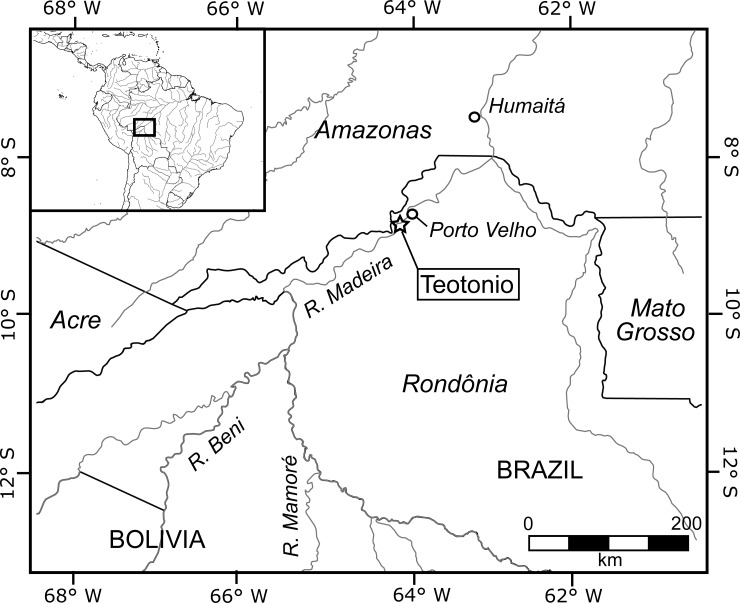
Map showing the location of the upper Madeira region and Teotonio site in southwestern Amazonia.

The Madeira river is the fourth largest in the world in terms of fluvial discharge [7]. It forms from the meeting of the Mamoré and Beni rivers, which both have headwaters high in the Bolivian Central Andes, and eventually joins the Amazon river some 1,000 km to the northeast. The Madeira is a classic Amazonian white-water river, being rich in nutrients carried downstream from the catchment areas of its upper tributaries. The upper Madeira region is defined as the stretch of this river between the meeting of the Mamoré and Beni rivers and the city of Humaitá 400 km downstream (Fig 1). It is characterized by the presence of rapids and waterfalls that are among the most voluminous in the world [7].

The upper Madeira has a humid tropical climate, with annual precipitation of 2,250–2750 mm/year, a short, three-month dry season between June and August, and mean annul temperatures of 24–26°C [8]. Vegetation has been highly impacted by human land use since the 1970s and, where it survives, consists mainly of open ombrophilous forest, interspersed with patches of dense ombriphilous forest, edaphically-restricted *campinarana* vegetation (wooded to open grassland), and alluvial (*várzea*) forest along the narrow river banks [9]. To the north of the study area, around the city of Humaitá, natural savanna formations cover an area of roughly 615 km^2^ [10]

The basic chronology and cultural sequence for southwestern Amazonia was established by Eurico Miller [6]. Doing fieldwork virtually on his own in the 1970s and 1980s, a time when the region began to suffer massive deforestation and colonization, Miller established a sequence of almost continuous occupation that started in the early Holocene, around 9,000 years BP, and continued until the time of the rubber boom in the early twentieth century. Despite dramatic depopulation in the last decades, southwestern Amazonia is home to distinct indigenous populations and one of the areas of highest language diversity in the world [11]. Many such populations live at the western edge of the so-called Amazonian deforestation arc and undergo diverse pressures on their lands. Nowadays, indigenous lands and conservation units are the only areas where extensive deforestation has not yet taken place.

In the last decade, a combination of academic and contract archaeology projects has recorded dozens more archaeological sites along the Madeira river and its tributaries [12–15], several of these have yielding pre-ceramic occupations. Although Miller’s terminal Pleistocene Piriquitos complex is yet to be re-confirmed, at least five sites have evidenced early Holocene occupations associated to the Girau phase [6], which are associated with radiocarbon dates of 9,524–9,400 cal. BP from the Teotonio site and 9,910–9,550 cal. BP from the Vista Alegre 1 site [15]. New radiocarbon dates associated with Massangana-phase deposits of lithic artefacts in what appear to be Anthropogenic Dark Earths, or terra preta matrices at Garbin and Teotônio sites have also pushed regional ADE formation as far back as ca. 7,000–6,790 to 8,600–8,420 cal. BP [13,15] and 6,495–6,400 cal. BP [16], respectively, which makes the dark earths of the Upper Madeira some 3,500 years older than in the rest of Amazonia.

### Teotonio site

**Fig 2:**
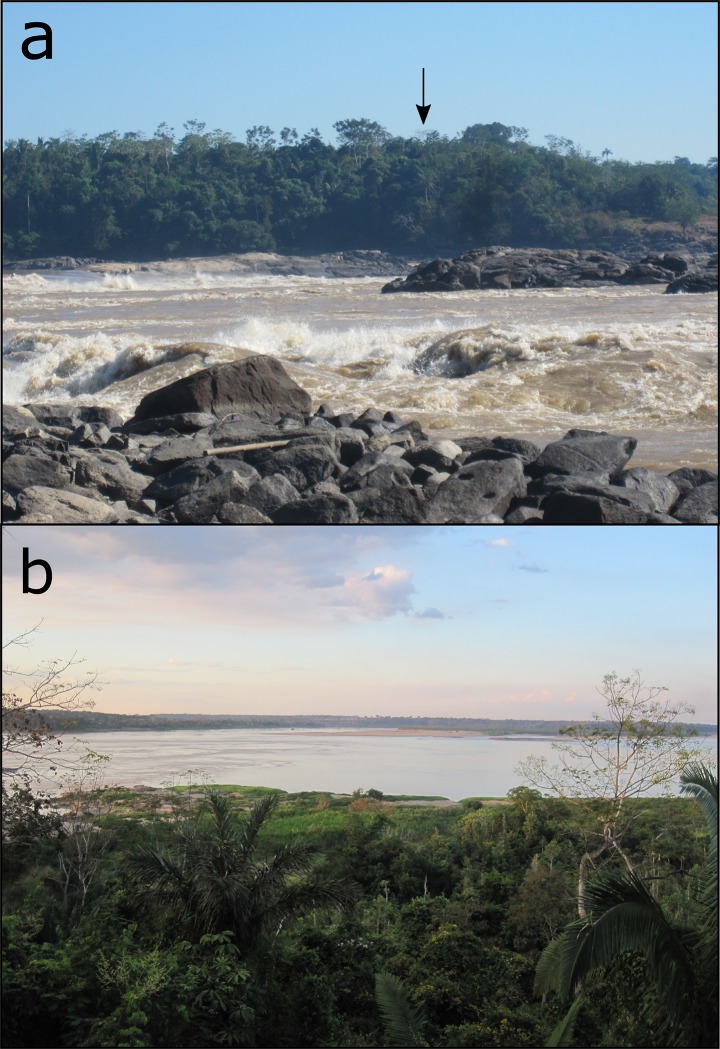
a) The *Cachoeira do Teotonio* viewed from the left bank of the Madeira river, with the location of Teotonio site indicated atop the river bluff; b) View of the Madeira river from Teotonio site. Both photographs were taken by E. Neves in 2011 before the construction of the Santo Antônio hydroelectric dam.

The Teotonio site (20L 383186/ 9020164) is situated on a 40 m-high river bluff on the right bank of the Madeira river adjacent to the *Cachoeira do Teotonio* (Fig 2), a set of rapids once famous for its abundance of fish [17] and, until the building of the Santo Antônio hydroelectric dam in 2013, the location of a thriving fishing village of the same name. The site has been subject to intermittent archaeological research since Miller’s first excavations there in the 1970s and more intensive excavations conducted by Arqueotrop (MAE, USP) since 2011 [12]. Almeida and Kater [18] liken Teotonio to a microcosm of human occupation of the Upper Madeira, since its archaeological sequence contains a record of almost every major regional cultural complex found in the area from the early Holocene onwards. Apart from the Girau and Massangana pre-ceramic occupations, which are the focus of this paper, Teotonio also contains at least three consecutive ceramist occupations from ca. 3000 BP onwards (Pocó-Açutuba, Jamari and Jatuarana) [18,19]. The most recent of these (the Jatuarana phase) has been discussed as an exceptionally early manifestation of the Amazonian Polychrome Tradition–a ceramic style that was produced all over the Amazon basin at European Contact [12,20]. Over the course of roughly 6000 years, human occupation of Teotonio created over 50 ha of ADEs [16], the scale and complexity of which is only just beginning to be understood.

The pre-ceramic contexts at Teotonio were fully exposed for the first time in 2013 within an open area adjacent to a dirt road that once led to the Teotonio waterfall. Road opening removed the superficial deposits in this area but did not interfere with the buried strata, as was attested to by the presence of a whole ceramic vessel whose rim was exposed at the road surface (see section”On-site soils (phytoliths and macroremains)”).

The excavation of the vessel showed it to be wholly in-tact and buried within an ADE deposit absent in ceramics and abundant in lithics. Further excavation of this unit (N9882, E10022 [hereby referred to as Unit 5]) revealed a Massangana horizon 110 cm-deep overlying a further 30 cm of orange-brown ferralsol that also contained lithic artefacts, which was classified as belonging to the Girau phase [16]. A radiocarbon date obtained from charcoal belonging to a fire structure at the base of the Massangana ADE gave a date of 6,495–6,400 cal. BP.

In 2016, a 4 x 1m trench was excavated adjacent to Unit 5 (N9877 to N9880 E10022 [hereby Units 1–4, respectively]) to further investigate these pre-ceramic contexts and collect more samples for radiocarbon dating, lithic and archaeobotanical analyses. Here the Massangana ADEs were shallower (50 cm deep) and the underlying non-ADE soils of Girau phase directly overlay a lateritic bedrock outcrop. Despite more recent dates obtained from a ceramic sherd in Unit 2, and a charcoal fragment in Unit 1 (Table 1; see section 3.2), two *in-situ* Massangana fire structures yielded dates between 5,643 and 5,900 cal. BP. Charcoal associated with lithic material at the base of the Girau horizon in Unit 2 yielded a *terminus post-quem* of 9,524–9,400 cal. BP for this material, and provided the first definitive evidence that early-Holocene occupations have been exposed in this area of the site.

**Table 1:** List of AMS dates obtained from pre-ceramic contexts discussed in the paper.

Unit	Depth (cm)	PN	Material	Lab code	C14 age years BP (+/- 2σ error)	Calibrated age BP
1	20	PN 2005	Charcoal from in-situ hearth feature situated within Massangana ADE	Beta 482331	5040 +/- 30	5771–5643
2	22	PN 2428	Bulk sherd date from ceramic plotted in soil profile	Beta 482333	1780 +/- 30	1720–1575
1	30–40	PN 2007	Charcoal from in-situ hearth feature situated within Massangana ADE	Beta 474438	5080 +/- 30	5900–5708
1	50	PN 2027	Charcoal plotted in profile at the base of the Massangana ADE horizon	Beta 474439	1110 +/- 30	994–924
5	100–110	Te-1966	Charcoal from in-situ hearth feature at base of Massangana ADE	Beta 408414	5720 +/- 30	6495–6400
2	130	PN 2426	Charcoal plotted in profile at the base of the Girau horizon	Beta 474440	8460 +/- 30	9524–9400

Profile drawings of these contexts, and more detailed chronostratigraphic interpretations, are provided below alongside a discussion of sample provenience.

## Materials and methods

### Lithic residue analysis (starch and phytoliths)

#### Contexts

Lithic artefacts retrieved from Unit 5 were tested for phytoliths and starch grains with the assumption that some may have been used to process vegetal material. This unit contained over 1,400 lithic fragments in total (1,015 unipolar flakes, 107 bipolar flakes and 152 cores) and over half of these (n = 884) were encountered between 40–90 cm (within the Massangana ADE) [16]. Lithics from both the Massangana and Girau occupations at Teotonio are characterized by small (average 0.9 cm-long), unipolar flakes made of hyaline and milky quartz [16]. Starch grain analysis of similar artefacts from the Orinoco valley, originally thought to be manioc grater teeth, showed that they were used to process a range of different plants [21].

As a way of insuring that the tested artefacts were used in the past, only those displaying evidence of retouch or with prominent sharp edges were chosen for phytolith and starch grain analysis. Twenty-nine retouched artefacts were identified and analyzed, 23 from the Massangana horizon, and 6 from the Girau horizon of Unit 5.

#### Extraction and identification

Since the lithic artefacts had already been lightly washed and handled during quantification and identification, if was deemed of vital importance to control for potential contamination during phytolith and starch extraction.

Following the step-wise protocol explained in Pearsall [22], lithic artefacts were subject to two stages of extraction. During the first stage, the artefact was thoroughly cleaned and rinsed to remove any adhering contamination by submersing it within a beaker partly filled with distilled water and cleaning it with a toothbrush. This was named the “wet brush” (WB) sample. This adhering residue was then concentrated by repeated rounds of centrifugation (3000 rpm for 5 min) within a test tube until nothing was left in the beakers. The tougher adhering residues, more likely to have arrived from the initial use of the artefacts, were extracted by placing them in an ultrasonic bath of distilled water for 5 mins and concentrating the resulting solution as before (the “ultrasonic” [US] sample).

Sample contamination during lab work was avoided by sterilising all laboratory equipment in an autoclave between uses (beakers, toothbrushes, test tubes, pipette tips, microscope slides, cover slips, etc.) and by handling the artefacts with tweezers (also sterilized). The use of latex gloves was avoided following studies that found maize starch even in non-powdered varieties [23].

To mount the residues for analysis, ~ 50 μl of the residue/distilled water solution was transferred onto the centre of a glass microscope slide and placed to dry in an oven at 38°C (greater than 40°C and the starch grains would start to gelatinize). Once dry, a drop of 1:1 glycerine:distilled water solution was placed on the slide and mixed thoroughly with the residue before adding a glass cover slip to seal. Slides were scanned in their entirety at 500 x magnification and photographed using Leica LAS 15 software.

Phytoliths and starch grains were identified and described using a range of published work from the Neotropics and elsewhere (e.g. [24–40]) and by comparison with a modern reference collection that includes over 100 species native to the Upper Madeira region. In some cases, starch grains exhibited damage from heating or grinding, and these were identified via comparisons with published works (e.g. [31,32]).

### On-site soils (phytoliths and macroremains)

#### Contexts

**Fig 3:**
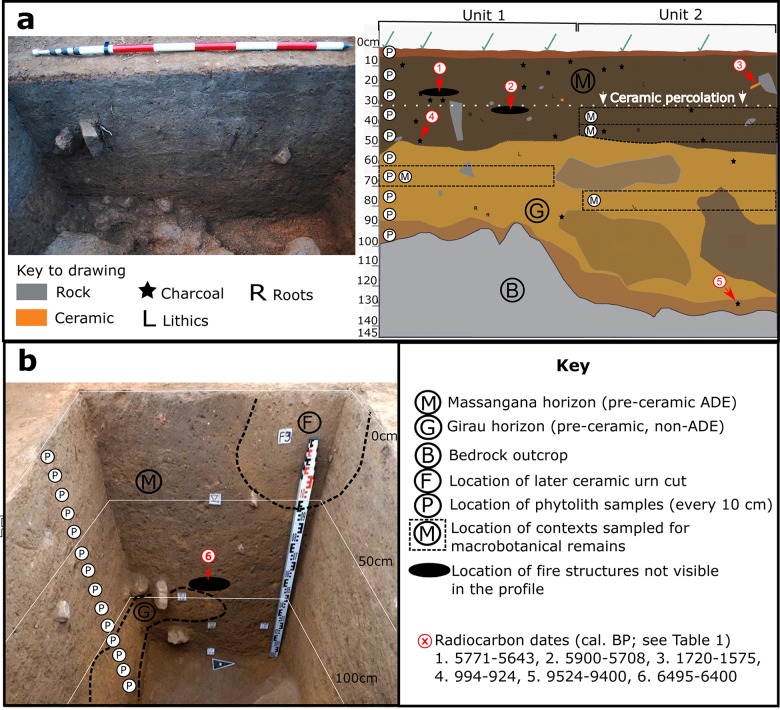
Images of excavated pre-ceramic units at Teotonio highlighting their stratigraphy, the location of radiocarbon dates, and the origin of archaeobotanical samples. a) Photograph and drawing of east-facing profiles of Units 1 and 2 (Arqueotrop, MAE, 2016), b) Photograph of east-facing profile of Unit 5 (Arqueotrop, MAE, 2011), with the relevant stratigraphic information projected upon it. Phytolith samples were taken from the north-facing profile.

Sediment samples for phytolith analysis were collected from the post-excavation profile walls of Unit 1 and 5 (Fig 3A and 3B). Samples weighing ca. 300 g were taken in 10 cm intervals, respecting the natural stratigraphy–for example, if the interface of natural levels *x* and *y* was at 18 cm below surface, sample 10–20 cm would comprise of material from level *x* only (10–18 cm).

A total of 80 litres of sediment were collected for the flotation of macrobotanical remains from Units 1 and 2, consisting of two 10-litre collections (parts 1 and 2) from each sampled context. The samples were set aside during archaeological excavation of the following levels: Massangana: Unit 2 (30–40 cm [20 litres] and 40–50 cm [20 litres]; Girau: Unit 1 (60–70 cm [20 litres]) and Unit 2 (70–80 m [20 litres]) (Fig 3A).

#### Stratigraphy

The presence of small quantities of ceramic fragments in the uppermost portion of the Massangana ADEs deposits was observed during fieldwork in 2016 and quantified during post-excavation. Mostly represented by very small fragments, and belonging to a previously-unseen technology, the question was raised as to whether we had found a late Massangana ceramic technology. Comparison of a bulk sherd date (994–924 cal. BP [Unit 2, 22 cm]) with that of a fire structure at the same depth (~ 5700 cal. BP [Unit 1, 20 cm]) demonstrates, however, that these ceramics were incorporated into the stratigraphy afterwards, perhaps from a later overlying occupation sequence that has been truncated by road-building.

Meanwhile, the age inversion introduced by charcoal from the base of the Massangana ADE (1720–1575 cal. BP [Unit 1, 50 cm]) may also be a product of post-depositional mixing, or the incorporation of burnt root material in the soil profile. Either way, as this charcoal fragment was floating in the profile, rather than part of a consolidated fire structure, we base chrono-stratigraphic interpretations on the former only.

Although there is evidence that post-depositional processes may have incorporated some younger material into the pre-ceramic sediment sequences under study, we do not believe them to have been severe enough to call into question the age of the associated archaeobotanical remains. In Unit 2, a total of 63 ceramic fragments, weighing just 178 g, were recovered in the top 30 cm of the Massangana ADE stratum (save for a singular fragment recovered in the 70–80 cm level) [16]. Level 20–30 cm yielded considerably less (8 fragments) than the superior levels, showing that the majority of post-depositional mixing occurred in the top 20 cm of the profile. Sediments for macrobotanical recovery were sourced below 30 cm (30–50 and 70–80 cm).

Ceramics are also confined to the top 30 cm of the profile of Unit 1, so we can expect older and younger phytoliths to be mixed in this portion of the profile, with the effect becoming less with depth. The only macrobotanical sample taken from this unit was from 60–70 cm depth.

#### Extraction and identification

Phytoliths were extracted from sediments using the wet oxidation method described by Piperno [24]. One hundred millilitres of sample was mixed with hot water and sodium hexametaphosphate and agitated for 24 h for deflocculation. Clays were removed by gravity sedimentation and the sample sieved into silt (< 53 μm) and sand (53–250 μm) fractions in order to concentrate large, diagnostic phytoliths such as those produced by squash and various Marantaceae tubers in the latter [24,33,34]. Hydrochloric acid (37%) was then used to remove carbonates and Nitric acid (60%), heated to 100 °C, to remove organics. Potassium chlorate was added to samples heated in the Nitric acid to aid the reaction. The Massangana ADEs were extremely rich in organic material and had to be left for up to four days in the Nitric acid stage, with the acid being changed every 8 hours. Phytoliths were floated from the soils using Zinc iodide heavy liquid prepared to a specific gravity of 2.3 g/cm^3^, before being treated with Acetone and left to dry for 24 hours. Permount mounting medium was used to allow the three-dimensional rotation of phytoliths during analysis.

The silt fraction was analyzed under 500 x magnification and an initial count of 200 phytoliths was performed on each sample. Since palm phytoliths dominated almost all the assemblages in Unit 1, it was decided to perform an extended count to reach 200 non-palm phytoliths to identify subtler changes in the assemblages that may have otherwise been missed; for means of comparison, this counting methodology was also performed on the Unit 5 samples. Once the extended count was reached, the rest of the slide was scanned for any additional taxa. All of the phytoliths present in the sand-fraction slides were counted and scanned under 200 × magnification. In both cases, only those phytoliths with some taxonomic significance were recorded. Phytolith identification followed published literature and comparisons with the phytolith reference collection of modern plants, housed in the Institute of Geosciences, University of São Paulo. Differentiation of Arecaceae phytoliths followed a recently-published reference collection of Amazonian palms [35]. Raw counts were converted into percentage frequencies and graphs produced using C2 software [36]. As with artefact analyses, Leica LAS 15 image capturing software was used.

Recovery of macrobotanical remains took place in the University of São Paulo using a floatation tank adapted by Shock for low water pressure from the SMAP flotation system [22]. Filter sizes of an outflow geological 0.5 mm sieve and an internal 1.5 mm removable wire mesh were used to capture the maximum range of plant remains. These samples were designated as the light and heavy fraction, respectively. Soil volume was confirmed prior to flotation.

Light fraction sample sorting involved the removal of only seed and fruit fragments with characteristic attributes due to the diminutive size of the material; very little charcoal was buoyant. Sorting of the light fraction was conducted under a stereomicroscope with 7–50x magnification. The heavy fraction material was standardized to 2 mm using a geological sieve to insure equitability and charcoal was manually separated from other cultural material and rock fragments.

Morphological and anatomical characteristics were employed to separate between wood, non-wood, and non-identifiable charcoal. The latter category was made necessary by the diminutive size of the charcoal and the numerous pieces with surface erosion and damage. Wood charcoal was not analyzed, and only non-wood charcoal with diagnostic or potentially diagnostic structures were separated for identification. Non-wood charcoal which could not be taxonomically identified were designated as “diagnostic”, meaning that they may be identifiable at a future date with more extensive reference collections. Identification was made possible using a modern reference collection of over 350 accessions of over 100 useful Amazonian species, housed at the Federal University of Western Pará, Santarém, Brazil.

## Results

### Artefact residues

**Fig 4:**
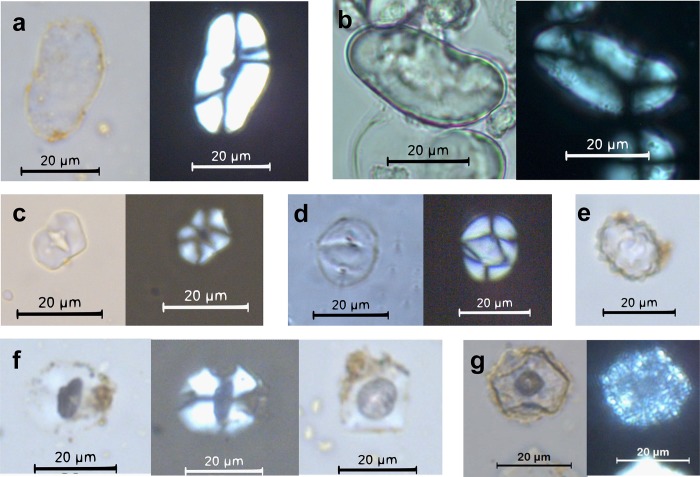
Photographs of starch grains encountered in lithic residues. a) *Phaseolus* sp. starch grain (artefact 1957–1 [US], Unit 5, 60–70 cm); b) Modern *Phaseolus vulgaris* starch grain; c) cf. Arecaceae starch (artefact 1972–1 [WB], Unit 5, 120–130 cm); d) Modern starch grain from *Attalea maripa* seed. This morphotype was also reported in other Arecaceae species in the comparative reference collection; e) Arecaceae phytolith (artefact 1974–1 [WB], Unit 5, 140–150 cm); f) Unidentified Type 1 starch grain showing darkened centre (hilum projections), rotated to show quadrangular face (1974–1 [US], Unit 5, 140–150 cm); g) cf. Unidentified Type 1 with shattered appearance under polarized light (1956–2 [US], Unit 5, 40–50 cm).

Eleven starch grain morphotypes were found in 14 lithic artefacts belonging to the Massangana (M) and Girau (G) contexts at Teotonio (Table 2). Of these 11 types, five were found exclusively in the WB samples and thus cannot be treated as not having derived from the original use of the artefact. These include the cf. Arecaceae-type starches found adhering to artefacts 1957–4 (M) and 1972–1 (G). These are compound grains consisting of two hemispherical starches with angular margins. The marginal position of their hila means that they sometimes appear as forming a singular extinction cross under polarized light (Fig 4C). They were identified in the seeds of several palm species in our modern reference collection (Fig 4D).

**Table 2:** Table showing starch grain and phytoliths encountered in Massangana and Girau residues from Unit 5.

			Starch grain frequencies						Phytolith presence			
	Level (cm BS)	Artefact no.	*Phaseolus* sp.	cf. Arecaceae	UID	UID types	Damage	TOTAL	Poaceae	Arecaceae	Arboreal	UID
**Massangana**	10–20	1951–1			[3]	[1, 2, 3]	HP	**3**			x	x
	20–30	1952–1						**0**		x		x
	20–30	1952–2						**0**				x
	20–30	1952–3						**0**				
	20–30	1952–4			[3]	[3, 4, 5]	HP	**3**				x
	20–30	1952–5			[1]	[6]		**1**		x*		
	30–40	1955–1			2	5		**2**			x	x
	30–40	1955–2			2	1	HP	**2**				
	30–40	1955–4						**0**			x	x
	30–40	1955–3						**0**				
	30–40	1955–5						**0**				x
	40–50	1956–4			6	1, 7, 8	HP, Sh, Enz?	**6**		x	x	x
	40–50	1956–2			4	1	HP, Sh	**4**		x	x	
	50–60	1957–1	1		2 [1]	1	HP	**4**		x [x]	x [x]	x
	50–60	1957–3	1		3	1	HP	**4**			x	x
	50–60	1957–4		[2]				**2**				x
	50–60	1957–5						**0**		[x]	x [x]	x [x]
	60–70	1958–1	1		3 [1]	1, [9]	HP	**5**		x [x]	x	x
	60–70	1958–2						**0**		x	x	x
	60–70	1958–3						**0**	x	x [x]	x	x
	80–90	1961–1			5 [2]	6 [1, 2, 6]	HP	**7**				x
	80–90	1962–2						**3**		x [x]		x
	90–100	1967–1						**0**	x	x	[x]	x [x]
	Fire structure	1971–1			[14]	[1, 3]	HP	**14**		[x]	x	[x]
	30–40	904–1						**0**				
	40–50	1005–1						**0**				
	50–60	1107–1						**0**				
	50–60	1206–1						**0**				
**Girau**	120–130	1972–1		[1]	[5]	[1, 6]	HP	**6**	[x]	x [x]	x [x]	x [x]
	120–130	1972–2						**0**	[x]	x [x]	[x]	
	130–140	1973–1						**0**		x [x]	x [x]	x [x]
	130–140	1973–2			1	6		**1**		x [x*]	x	x [x]
	130–140	1973–3			[10]	[1, 3]	HP	**10**	x	x [x]	x [x]	x [x]
	140–150	1974–1			3 [6]	1 [1, 3, 8]	HP	**9**		x [*]	[x]	[x]

UID = unidentified; [] = starches or phytoliths found in WB samples; Arecaceae phytoliths: x = globular echinates (in all palms except *Bactris/Astrocaryum* spp.), * = conical bodies (specific to *Bactris/Astrocaryum* spp.); Damage types: HP = hilum projections, Sh = shattered; Enz = enzymatic damage.

**Fig 5:**
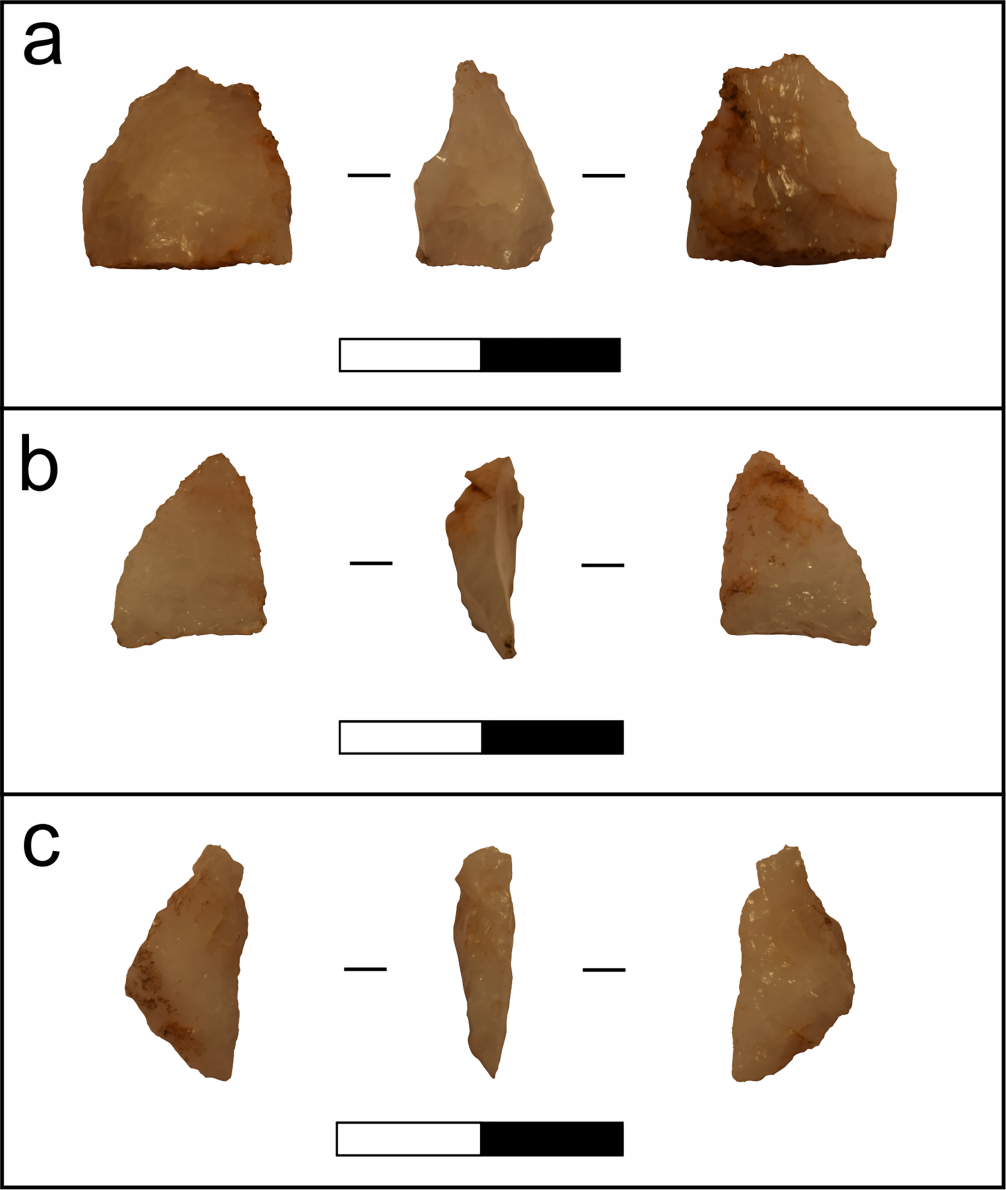
Photographs of lithic artefacts that yielded *Phaseolus* sp. starch grains. a) Artefact 1957–1: Unipolar flake with retouch on its distal portion; b) Artefact 1957–3: Unipolar flake with retouch on its right margin; c) Artefact 1958–1: Unipolar flake fragment with retouch on its right margin (taken from [16]). Scales = 2 cm.

Three starch grains pertaining to beans (*Phaseolus* sp., Fabaceae) were identified in US samples of three different Massangana artefacts found in the 50–60 cm and 60–70 cm excavation levels (1957–1, 1957–3 and 1958–1, Fig 5), and were the only confidently identifiable archaeological starch grains in the study. These grains were oval-shaped and laminated in front view and reniform in side view and measured ca. 30 μm long. The jagged longitudinal cleft characteristic of *Phaseolus* starch grains was not clearly visible under transmitted light but could be inferred by the form of the extinction cross under polarized light (Fig 4A). The fact that none of these starches were present in the WB samples from the same artefacts implies that they derive from the original use of these tools rather than modern contamination.

Of the unidentified starch grains encountered in the US samples, Type 1 (Fig 4E) deserves special mention, since it was encountered in residues from 11 different (M and G) artefacts. This morphotype is pentagonal in front view and quadrangular in side view, with rounded margins, a slightly curved extinction cross, and an open hilum, and its typical maximum length is between 10–20 μm. In the majority of cases, there was an area of discolouration in the centre of the grain which also showed as a darkened centre under polarized light. According to the International Code for Starch Nomenclature (ICSN) [37], these dark areas may be “hilum projections” (HP) that occur as an effect of roasting (see also [31]). In artefacts 1956–2 and 1956–4, we also recovered starch grains with the same overall size and shape (pentagonal to quadrangular) and the same darkened centre, however these displayed more angular margins and a shattered appearance (Sh) under polarized light (Fig 4F). We tentatively identify these as Type 1 starches that have been subject to further unknown taphonomic processes. This evidence suggests that several of the quartz flakes were used with a specific plant species that had already undergone processing.

Phytolith assemblages from the lithic artefacts were dominated by two morphotypes, which were encountered together in the majority of analyzed pieces. The “woody” category in Table 2 refers to globular granulate phytoliths, produced in the wood of tropical trees and shrubs [38,39], and these had a similar distribution to palm phytoliths. Palm species that produce conical phytoliths (*Bactris/Astrocaryum* spp.) were present in much rarer quantities than those producing globular echinate phytoliths (all other species). Considering that arboreal and palms make up the majority of the soil phytolith assemblages from the same unit (see next section), it is most likely that the phytoliths from the lithic residues represent background vegetation as opposed to original use of the tools. The rarer presence of Panicoideae grasses (bilobates) and Bambusoideae phytoliths (tall/collapsed saddles) in the residues and soil samples supports this interpretation.

On the other hand, two unidentified phytolith types were encountered in lithic residues that were not present in the surrounding soil samples. The UID 1 morphotype was found on 7 tools (5 in US and 2 in WB samples) and is a silicified hair base, most likely deriving from a dicotyledonous plant. UID 4 was found in the US samples of two artefacts (1957–1 (M) and 1961–1 (G)) and is a sphere with verrucate surface decoration approximately 20 μm in diameter, of unknown origin. These phytoliths may have originated from plants processed using these artefacts, but we are unable to say for sure.

### On-site soils

#### Phytolith results

**Fig 6:**
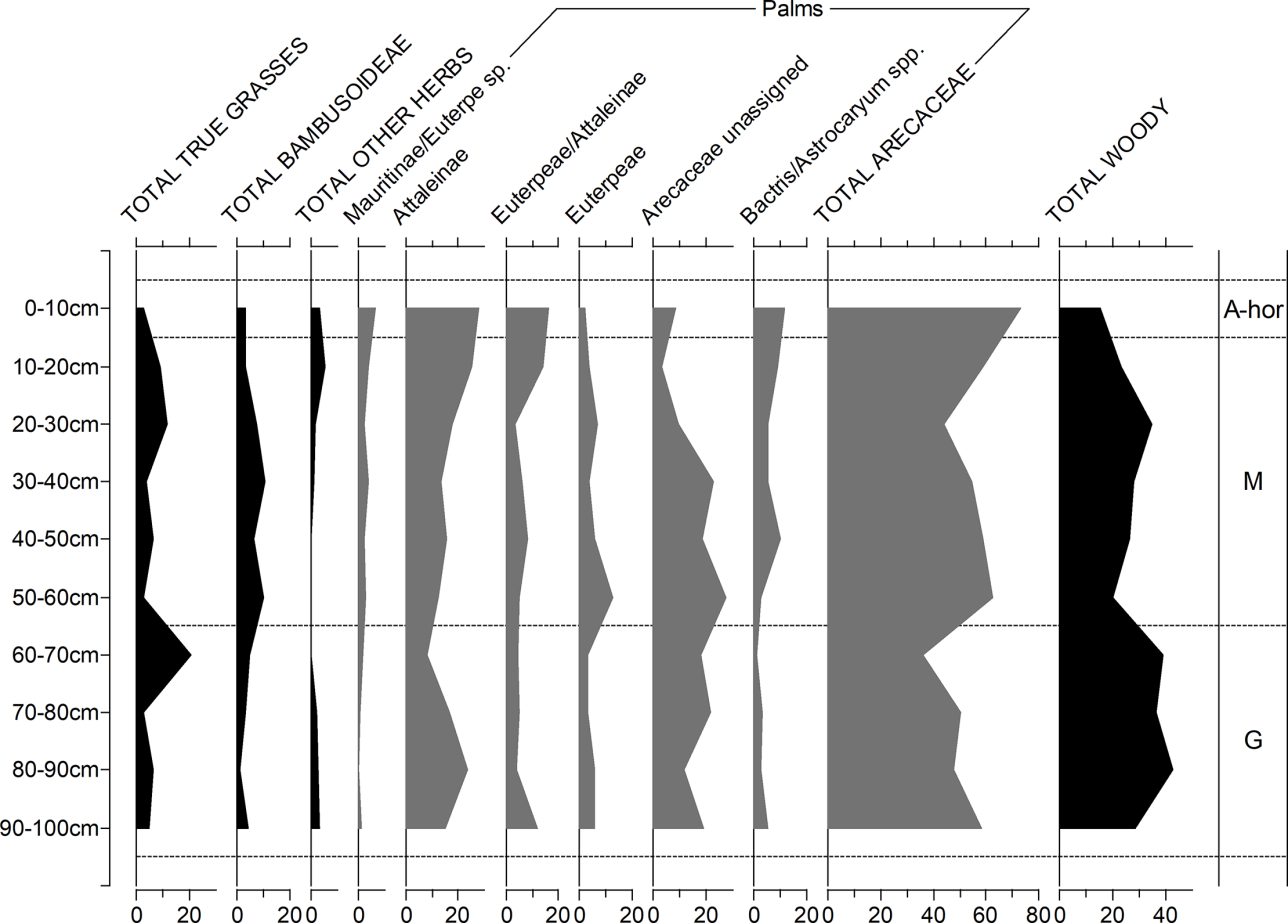
Relative frequency diagram of phytoliths from Unit 1 showing main taxonomic groups and composition of palm counts (in grey). A-hor = A horizon, M = Massangana, G = Girau.

**Fig 7:**
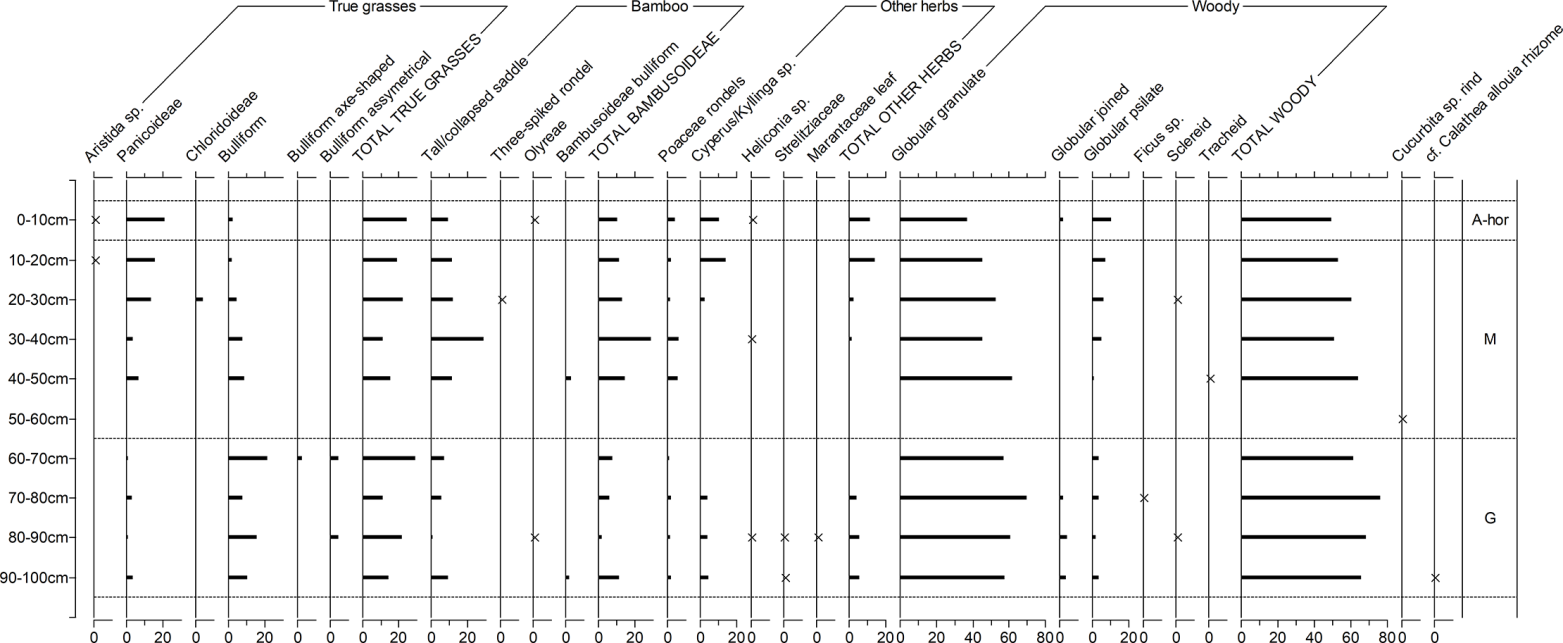
Relative frequency diagram of phytoliths from Unit 5 showing main taxonomic groups and composition of palm counts (in grey). A-hor = A horizon, M = Massangana, G = Girau.

**Fig 8:**
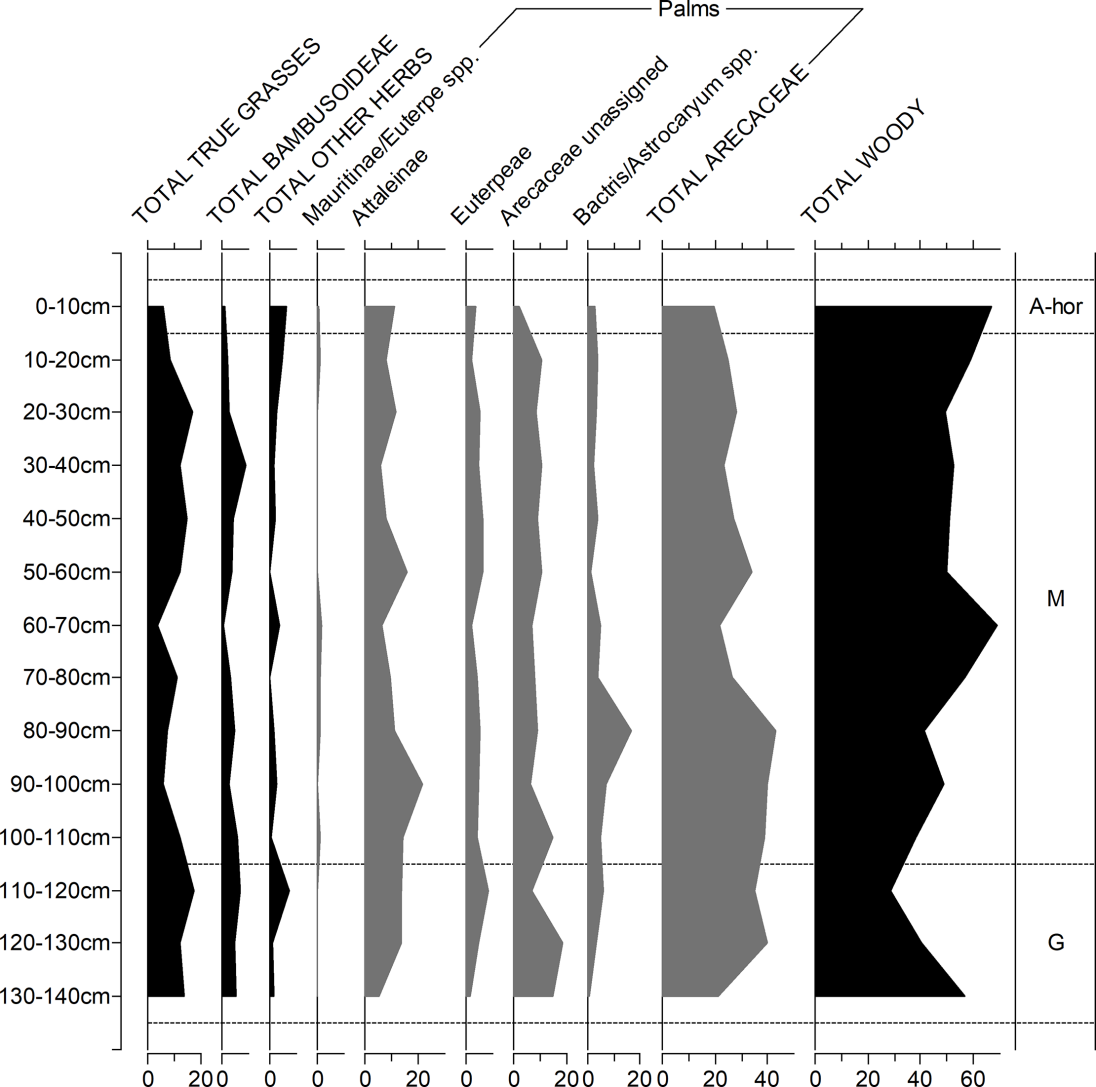
Relative frequency diagram of extended phytolith (non-palm) counts in Unit 1. A-hor = A horizon, M = Massangana, G = Girau. Crosses are used instead of bars to highlight taxa with low frequencies.

**Fig 9:**
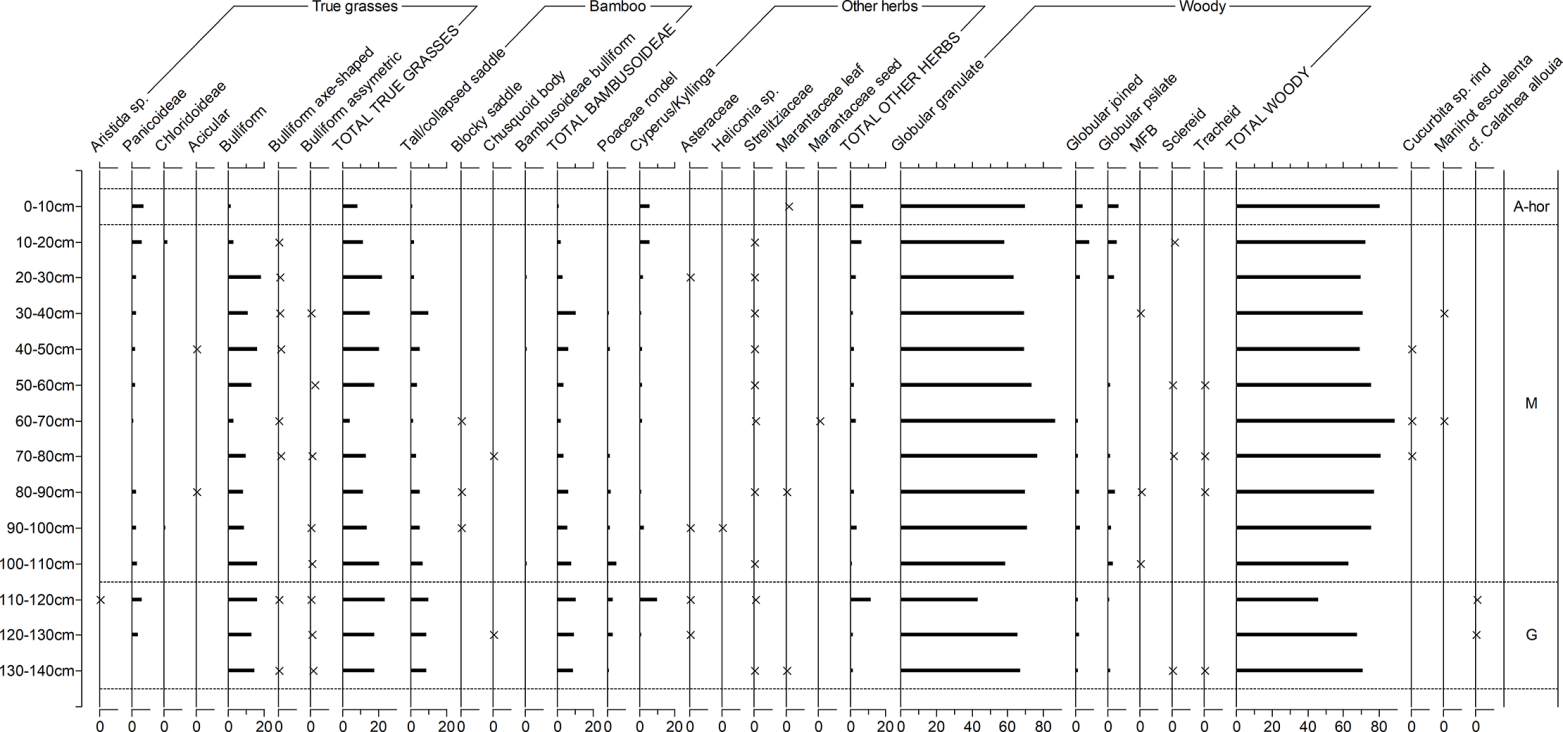
Relative frequency diagram of extended phytolith (non-palm) counts in Unit 5. A-hor = A-horizon, M = Massangana, G = Girau. Crosses are used instead of bars to highlight taxa with low frequencies.

Figs 6 to 9 depict the phytolith results from the on-site soils. Figs 6 and 7 are simplified counts showing the main taxonomic groups and all palm phytolith types. Figs 8 and 9 show the results of the extended counts (without palm phytoliths).

**A horizons (0–10 cm).** In Unit 1, palms make up nearly 80% of the assemblage from the A horizon, with globular echinate types pertaining to Attaleinae (*Attalea* sp.) palms being the most abundant. *Attalea* spp. are common disturbance indicators, alongside *Cyperus* sp., *Heliconia* sp. and Panicoideae grasses, which also peak in this part of the profile.

A slight increase in *Cyperus* sp. and Panicoideae grasses is also seen in the A-horizon of Unit 5, however here, globular granulate phytoliths from arboreal species dominate the counts (~ 60%) while Attaleinae palms constitute only 10%. The fact that this disparity in the A-horizon phytolith assemblages continues throughout the entirety of both sequences leads us to argue that, rather than reflecting mostly modern vegetation, the surface phytoliths are already reflecting the archaeological contexts. This scenario is likely a result of the building of the recent road, which likely truncated and sealed the archaeological record in this area of the site.

**Fig 10:**
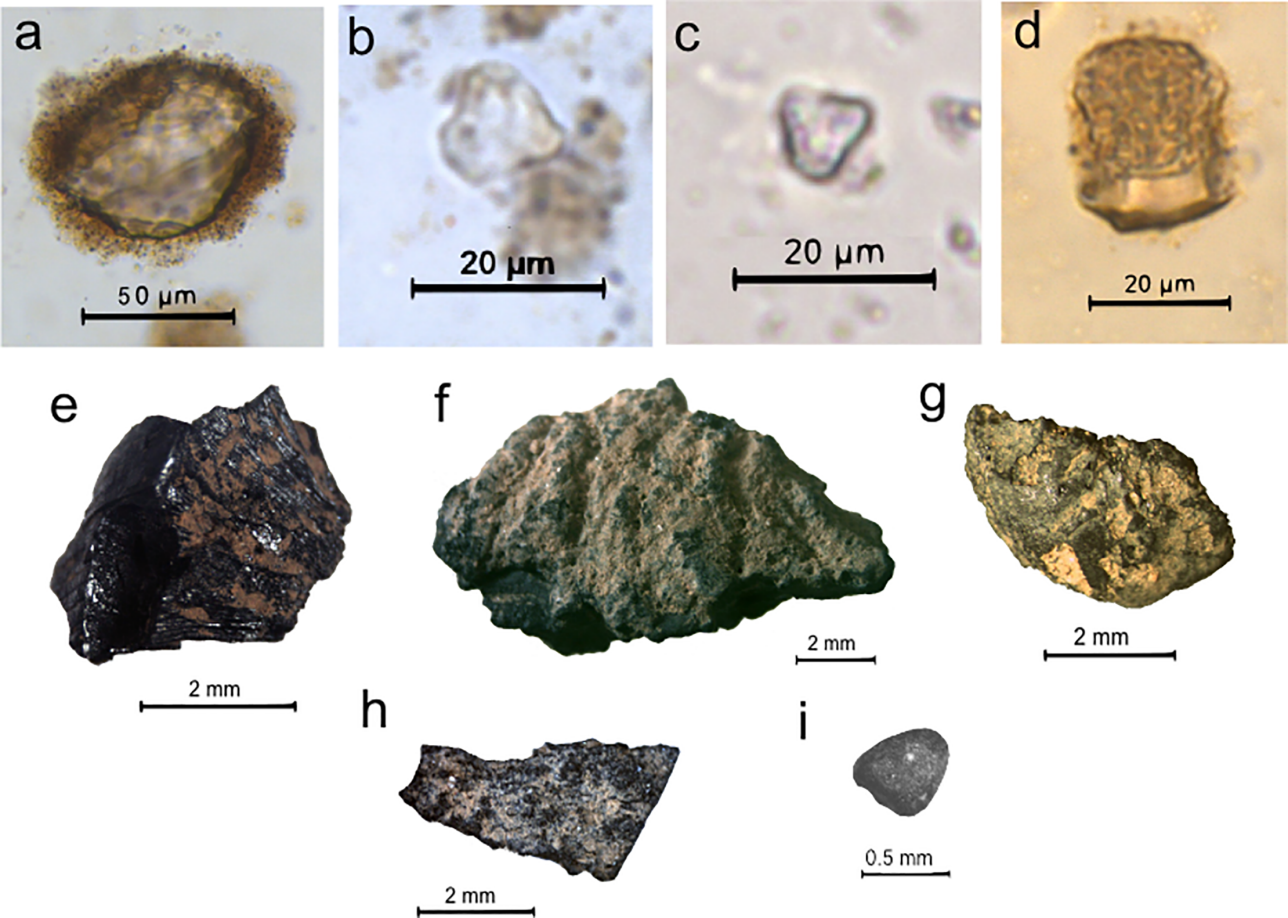
Images of selected phytoliths and macroremains encountered in pre-ceramic on-site soils at Teotonio. a) *Cucurbita* sp. phytolith (Unit 1, 50–60 cm); b) *Manihot esculenta* phytolith (Unit 5, 60–70 cm); c) Modern *M*. *esculenta* phytolith extracted from root rind material; d) cf. *Calathea allouia* phytolith; e) *Oenocarpus* sp. seed fragment (Unit 2, 40–50 cm); f) *Bertholletia* excelsa seed case fragment; g) *Phaseolus/Vigna* sp. bean (Unit 1, 60–70 cm); h) *Caryocar* sp. seed fragment (Unit 2, 70–80 cm); i) *Psidium* sp. seed (Unit 2, 70–80 cm).

**Massangana (Unit 1, 10–60 cm; Unit 5, 10–110 cm).** In the Unit 1 Massangana sequence, palms range between 50–65% and arboreal phytoliths between 20–35%, with the rest made up of grasses and herbs. A marked change in the phytolith record, however, seems to occur below 30 cm, whereby Attaleinae palms and other disturbance indicators (Panicoideae grasses and *Cyperus* sp.) swiftly decline, to be replaced by Euterpeae (*Euterpe/Oenocarpus* spp.) palms, grass bulliform phytoliths, and a large peak in bamboo (Bambusoideae) leaf phytoliths (>20% of the extended count). This transition occurs in the same level as one of the dated fire structures (Table 1) and is accompanied by a substantial dip in overall phytolith recovery that continues until the base of the Massangana ADE. In the 50–60 cm sample, only 40 silt fraction phytoliths were recovered on the whole silt fraction slide. Since this is not a statistically significant quantity, phytolith data for this level are left blank in Fig 6. In the sand fraction of the same sample, however, was recovered one scalloped sphere phytolith from the rind of squash (*Cucurbita* sp.) (Fig 10A). The length and thickness of the squash phytolith (74 μm and 48 μm respectively) are within the range of domesticated *Cucurbita* species [40].

The Massangana phytolith assemblages of Unit 5 differ in several ways. Palm phytoliths continue to be less abundant than in Unit 1 (20–40%), although the groups represented remain similar (largely Attaleinae and Euterpeae, and a smaller quantity of Bactrinae). Arboreal taxa dominate the initial and extended counts count, while grasses and herbs are less than 20%. Bamboo leaf phytoliths are present but less abundant than Unit 1 (average = 6%, compared to 17%).

Manioc phytoliths (one per sample) were identified in levels 30–40 and 60–70 cm of Unit 5 (Fig 10B). These heart-shaped morphotypes are silicified excretory cells which are produced rarely in the root rinds, leaves, stems and fruits of *Manihot esculenta* [28]. Their maximum lengths were 11.3 μm and 12 μm which are in the upper size range reported for these phytoliths (5–12 μm, [28]). Three squash phytoliths were also recovered between 40–50 and 60–80 cm. The lengths and thicknesses of two of these (91 x 65 μm and 90 x 80 μm) again fall into the domesticated range, but the third was much smaller at 63 x 42 μm. Although domesticated *Cucurbita* species can produce phytoliths this small, these dimensions fit better into the mean ranges for wild *Cucurbita* (14–72 μm x 31–59 μm) [40]. Our sample size is, however, too small to suggest the presence of non-domesticated squash.

**Girau (Unit 1, 60–100 cm; Unit 5, 110–140 cm).** In Unit 1, palms phytoliths are relatively less abundant in the Girau levels than in the Massangana, making up 35–50% of the initial count, and this is offset by an increase in arboreal phytoliths. The majority of the globular echinate palm phytoliths were too eroded to be able to assign them to specific tribes, however Attaleinae palms phytoliths increased threefold between 60–90 cm (8–25%). This is accompanied by an increase in herbaceous disturbance indicators, including Bambusoideae, Olyreae, *Cyperus* sp., *Heliconia* sp., Strelitziaceae and Marantaceae in the basal samples.

In the extended Girau counts, grass frequencies are driven almost exclusively by bulliform phytoliths, which increase as Panicoideae grass short cells fall to very low numbers (<1%). Two different (non-cuneiform) bulliform morphologies (“axe-shaped” and “asymmetrical”) also appear for the first time in the Girau phytolith assemblages, suggesting a change in what types of grasses were present at this time.

Phytolith assemblages from the Girau horizon of Unit 5 are more similar to those from Unit 1. Sample 110–120 cm contained the highest frequency of disturbance indicators of the whole profile, as Panicoideae grasses and *Cyperus* sp. both peak to 6% and Strelitziaceae and Asteraceae make a presence. Disturbance indicators and Attaleinaea palms, however, suddenly drop off at 130–140 cm. Since only three lithic artefacts were recovered from this level (compared to 57 in the one above), this pattern could represent the transition to the natural ferralsol.

The differences in Panicoideae and bulliform phytolith frequencies seen in Unit 1 is less marked in Unit 5. Although assymetrical bulliforms are more frequent towards the base of the profile, bulliforms in general are as abundant in the Massangana as in the Girau horizon.

Particularly noteworthy in both units is the presence of phytoliths belonging to *Calathea* sp. and, perhaps (cf.), *C*. *allouia*. Known as leren, *C*. *allouia* is a species of domesticated tuber belonging to the Marantaceae family, and it has been identified from various early food production sites in both central and south America based on the type of phytolith we recovered at Teotonio (see Discussion). This phytolith pertains to the flat-domed cylinder type produced in the rhizomes (i.e. the edible part) of the plant (Fig 10D) and consists of a cylindrical body with ciliate decoration and a flat, smooth head which is polygonal in cross-section [28]. This morphotype can occur in some other *Calathea* species, but they are usually smaller and rougher than those which we encountered (which measured 26, 35 and 38 μm -long). Since around a dozen species of *Calathea* (syn. *Goeppertia* sp.) have been recorded in the Upper Madeira region, and not all have been tested for phytoliths, we propose a tentative identification.

#### Macrobotanical results

A total of 2,932 charcoal fragments were recovered from the heavy fractions, and 183 non-wood charcoals with diagnostic or potentially-diagnostic structures were separated for identification from the heavy and light fractions altogether.

**Fig 11:**
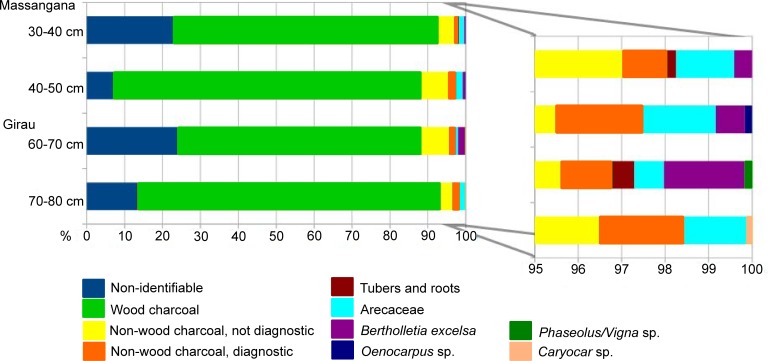
Graph showing relative frequencies of macrobotanical remains from the heavy fraction.

In sample 70–80 cm (part 2), seed coats from a single Poaceae species with brown/orange hues (incomplete or no charring) were identified and considered to be intrusive material from insect activity. Due to this potential contamination, all incompletely-charred material that was encountered in the light fractions was left out of the archaeological results (136 Poaceae; 30 other seeds). Results are depicted in Table 3 and Fig 11.

**Table 3:** Table showing frequencies of macrobotanical remains with diagnostic or potentially-diagnostic structures (absolute counts).

		Heavy fraction							Light fraction					
		Non-wood diagnostic charcoal (seeds and fruits)	Tubers and roots	Arecaceae	*Bertholletia excelsa*	*Vigna* sp. or *Phaseolus* sp.	*Caryocar* sp.	cf. *Oenocarpus* sp.	Diagnostic seeds	Diagnostic fruits	Tuber	*Psidum* sp.	Asteraceae	Piperaceae
Massangana	30–40 cm	10	2	13	4				2	3	2			
	40–50 cm	12		10	4			1	14	8				
Girau	60–70 cm	7	3	4	11	1			10	4				
	70–80 cm	15		11			1		18	8		1	3	1

**Massangana (Unit 2, 30–50 cm).** Wood charcoal constitutes the majority of macrobotanical remains recuperated from the Massangana ADE (70–81%), while seeds, fruits, tubers and roots account for less than 12%. Unidentified tuber/root fragments also appeared at 30–40 cm. Despite the fact that palms dominated the Massangana phytolith assemblages, only 23 fragments (1–2% of macrobotanical remains) were recovered. Of these, it was possible to separate the genus *Oenocarpus* sp. based on distinctive morphological characteristics (Fig 10E). Brazil nut (*Bertholletia excelsa*) seed coats were also recovered in trace amounts (<1%) (Fig 10F) and a potential *Manihot* fruit capsule recovered at 30–40 cm.

**Girau (Unit 1, 60–70 cm; Unit 2, 70–80 cm).** Wood charcoal again dominated macrobotanical counts from the Girau context (62–80%), and the proportions seeds, fruit, root and tuber remains are similar to the Massangana. Tuber/root fragments were recovered from sample 60–70 cm, along with the highest recovery of Brazil nut seeds of all the samples (2%) and a single bean fragment (Fig 10G). The morphology of the bean fragment shows it to belong to either *Vigna* sp. or *Phaseolus* sp.–if the latter, this would complement the findings of *Phaseolus* starch in Massangana lithic residues. The 70–80 cm sample from the adjacent unit did not yield tuber fragments or Brazil nuts, and instead presented carbonized remains of *pequiá* (*Caryocar* sp.) (Fig 10H), guava (*Psidium sp*.) (Fig 10I) and Asteraceae seeds.

## Discussion

### Early and mid-Holocene resource use

#### The Girau phase: Pushing back the origins of plant cultivation

The discovery of *Calathea* sp. rhizome phytoliths in pre-ADE soils from the Girau phase is evidence for the cultivation of this genus at Teotonio in the early Holocene. The specific phytoliths we recovered have only been reported in one species (leren [*Calathea allouia*]); however, we maintain a tentative (cf. leren) identification since phytolith production is unknown for most native *Calathea* in our study area. Should its presence be confirmed in the future, it would add to other studies that document this crop–which was possibly domesticated in the seasonal forests of Central and northern South America [41]–in some of the earliest food production systems in the New World. The cylindrical flat dome phytoliths we recovered at Teotonio have been found in contexts dating to 8,600 cal. BP in the Aguadulce rock shelter in central Panama [3] and at the Peña Roja site in the Colombian Amazon dating to ca. 9,000 cal. BP [41]. In both cases, they were identified to leren (*C*. *allouia*) specifically. Recovery of these phytoliths at the Las Vegas site in SW Ecuador in 9,000 cal. BP contexts was also interpreted as representing the early presence of this crop [40], while its starch has also been found on grinding stones from the southern Ecuadorian highlands dated to 8,000 cal. BP [42].

The *Calathea* sp. phytoliths, and the presence of carbonized tuber/root fragments in the macrobotanical record, show that cultivated tubers and roots were part of early-mid Holocene diets at Teotonio. While manioc was absent from Girau contexts, the presence of manioc starch grains on stone tools dating to at least ca. 7,000 cal. BP in central Panama [43], the Rio Porce area of northwestern Colombia [44], and the Zaña Valley in the lower western slopes of the Peruvian Andes [45] implies that it was during the Girau–and not Massangana–period that manioc was first cultivated and eventually domesticated in the Upper Madeira before spreading across the Americas [46]—indeed, genetic evidence points to a domestication event sometime between 8,000 and 10,000 BP [4]. These studies provide circumstantial evidence for manioc cultivation during the Girau phase at Teotonio, and raise the possibility that its absence in our study might be due to insufficient sampling and/or its low visibility in the archaeobotanical record.

The presence of *pequiá*, guava and Brazil nuts in the Girau macrobotanical record implies that gathered fruits and nuts also played a key role in early Holocene subsistence economies. All three of these species are of widespread economic importance today in the Amazon region. Brazil nut is the most important non-timber forest product in terms of export value [47]. Carbonized Brazil nut seed coats were found in ca. 11,000 cal. BP deposits at Pedra Pintada cave, Monte Alegre, Pará [48], and genetic evidence shows this species to have been rapidly dispersed across Amazonia by human populations in the late Holocene [47]. Meanwhile, the *pequiá* (*Caryocar sp*.) seed coat identified at Teotonio might belong to *Caryocar villosum*, but this is unconfirmed. *C*. *villosum* is the most intensively-exploited *pequiá* today and probably had incipiently-domesticated populations in Amazonia at European contact [49], however,the presence of *C*. *glabrum* at Peña Roja between 8,000–9,000 BP [50,51] suggests that other *pequiá* species were also exploited during the early Holocene. Both Brazil nut and *pequiá* are evergreen *terra firme* forest species and are circumstantial evidence for the presence of humid forest near the Teotonio site during the early Holocene. Guava, on the other hand, is an invasive species with a strong preference for disturbed areas, and most likely had semi-domesticated populations in the past due to its intentional management by pre-Columbian populations [52]. Hence it is likely that the co-evolutionary process of anthropogenic forest disturbance and guava domestication was already underway during the Girau phase occupations at Teotonio.

#### The Massangana phase: Investing in cultivars

The macrobotanical remains show that Brazil nuts and palm fruits continued to be exploited during the Massangana phase and attest to the continued importance of gathered resources to the lifeways of these mid-Holocene populations. Perhaps it was other gathered and/or managed resources which contributed the seven unidentified starch grain types which were encountered in the Massangana lithic residues. One of these (type 1) was particularly ubiquitous and displayed damage consistent with roasting.

The discovery of manioc phytoliths in the Massangana ADEs from Unit 5 is exceptional because they are produced in very low quantities and are hard to find even in comparative modern reference material. We hypothesize that manioc phytoliths were incorporated into the ADEs of Unit 5 as by-products of its cultivation or processing. It is not the edible part of the root, but instead the root rind, leaves and fruits that produce phytoliths, and these had to have been discarded in reasonably high quantities to have been detected in the phytolith assemblages. The recovery of manioc phytoliths, which intriguingly coincides with the disappearance of *Calathea* sp. (cf. leren) phytoliths, neither proves nor disproves Arroyo-Kalin´s [53] hypothesis that increased sedentism demonstrated by ADE accumulation during the Massangana phase came about through an investment in manioc cultivation.

The absence of manioc starch grains in the 29 Massangana quartz flakes analyzed in this study rules these tools out as manioc grater teeth (*sensu* [21]). This should not come as a surprise, however, since bitter manioc varieties that have a widespread distribution today, and which are grated and soaked to remove cyagenous toxins before consumption, were selected by people much later [53–55]. It was instead the sweet variety, which requires only boiling or roasting to be edible, that was originally domesticated in southwest Amazonia and spread rapidly throughout the neotropics [46,54].

Massangana-age beans and squash at Teotonio is both the first and the earliest evidence of simultaneous cultivation of these two exotic crops in the Amazonian lowlands.

Squashes grow best in areas with moderate rainfall and a long dry season [41]. The squash phytoliths found at Teotonio do not permit identification to species level, however the two most likely contenders are *C*. *moschata* and *C*. *maxima*, the former being the most adapted to tropical hot and humid conditions [41]. *C*. *moschata* was cultivated in the Andes alongside native species *C*. *ecuadoriensis* by at least 10,000 cal. BP [25,40,56], making squashes one of the very earliest components of food production systems in South America. Positive identifications of *C*. *maxima* have not yet been made; however, Piperno [3] plots its tentative centre of origin either in the mid-elevation sub-Andes of Bolivia, or the Upper Madeira/Guaporé region. Our one very small squash phytolith was not enough to suggest the presence of non-fully domesticated *Cucurbita* at Teotonio, however it is intriguing that Rondônia is the state where cultivation of *C*. *maxima* is most widespread in Brazil today [57].

*Phaseolus* sp. is adapted to dry environments with intermediate temperatures in the mid-elevation neotropics [41]. An exotic crop to Amazonia, the starch grains identified on three Massangana lithic tools at Teotonio must have come from introduced domesticated species *P*. *vulgaris* (common bean) or *P*. *lunata* (lima bean). Genetic data suggest that common bean was independently domesticated in Mesoamerica (western-central Mexico/Guatemala) between 8,200–8,500 BP, and in the Andes (southern Peru/Bolivia) between 6,300–7,000 BP [41,58,59]. Intriguingly, the same two geographical centres are also where lima beans originate, although the domestication timings are less understood than for common bean [41,60,61].

At the time of contact, beans and squash were famously planted alongside maize across north and central America, and the cultivation of these three crops together is thought to have had very early origins [62–64]. They are found in various combinations in Andean sites as early as 8,000 cal. BP [25,42]. In the Amazon lowlands, squash and maize have been recovered together at Lake Rogaguado (Bolivia) and the Monte Castelo shell midden (Brazil), dating from ca. 6,000 and 6,500 cal. BP and ca. 5,200 cal. BP, respectively [65,66].

Could it be that beans and squash arrived together in the Upper Madeira during the Massangana phase? And could they also have been accompanied by maize? Whatever the case, the early adoption of these exotic cultivars is interesting in light of what we know about farmers´ uses of ADEs today.

### ADEs and food production

While manioc could have been cultivated successfully on the natural soils of the upper Madeira, yields of beans and squash would have almost certainly benefitted from the more fertile soils formed by the Massangana occupations–if these were indeed planted upon. Beans have very high phosphorous requirements that are seldom met in non-ADE soils, while the microfungi responsible for nitrogen fixation are so abundant in ADEs that leguminous plants often have a competitive advantage during fallow periods [67,68]. Modern farmers also report far better squash yields on ADE soils [69].

Studies into the modern use of ADEs continue to show their importance as areas for crop experimentation and demonstrate a strong correlation between ADEs and the planting of ‘exotic’ cultivars [68,70–72]. Since *Phaseolus* beans, and very likely squash, were exotic to southwest Amazonia, we hypothesize that ADE formation provided new and important micro-environments for experimenting with these cultivars, within what could be considered home garden-type settings [73–75]. We cannot rule out the possibility, however, that people also cultivated upon the fertile floodplain soils adjacent to the Madeira river.

### Landscape domestication before ADE formation

Due to the fairly shallow nature of the Girau occupation levels identified during archaeological excavations (~30 cm), and the limited spatial resolution represented by our archaeobotanical sampling locations, interpretations regarding landscape ecology should be regarded as preliminary.

The data gathered here suggests, however, that the beginnings of landscape domestication, at least on the site-level scale, began during the Girau phase (at least 9,500 cal. BP years ago), before ADEs began to be formed. This is argued through: 1) the presence of palms, particularly disturbance-loving *Attalea* sp., in equal or greater quantities than in Massangana levels (note that Miller [6] originally hypothesized that *Attalea* sp. became more abundant in the landscape during the Massangana–not the Girau–phase), 2) the presence of herbaceous disturbance indicators including Poaceae, *Cyperus* sp., *Heliconia* sp., Strelitziaceae and Asteraceae (including charred seeds of the latter), again in equal or greater quantities than in Massangana levels, 3) the presence of possible leren phytoliths, 4) the presence of guava, which is a secondary forest cultivar and 5) the absence of any “natural” phytolith assemblage above the bedrock encountered in Unit 1.

The fact that our modest number of samples imply landscape domestication–at least to some degree–during the Girau occupations, supports a scenario of great time-depth of human modifications to Amazonian ecosystems [48,76,77], in-line with other global tropical forest biomes [78].

The pervading view is that the early Holocene inhabitants of the upper Madeira were simple hunter-gatherers from a “pre”-landscape domestication age [6]. However, this scenario seems unlikely if we think about the following: we know that manioc was domesticated–and therefore cultivated–by societies in the same region during the Girau phase, and probably, we have argued, by the Girau-period inhabitants themselves. In domesticating manioc, people not only improved various aspects such as the size and production of its tubers, photosynthetic rates and seed functionality, but they did this by repeated cycles of recombination and selection which involved introducing clonal propagation as a viable reproductive mechanism (wild manioc cannot reproduce from stem cuttings) [5]. This process, which was completed by 8,000 years ago, required highly sophisticated knowledge of the natural world, and likely involved the manipulation of other aspects of the environment, including forest burning. Based on these considerations, it is also plausible that people were propagating and concentrating other useful plants within the wider landscape during this period.

That landscape domestication may have intensified during the Massangana phase is likely. ADE formation constituted an enduring form of landscape domestication that would have benefitted subsequent occupations by creating new niches with higher soil fertility and agrobiodiversity [79–81]. The beginnings of landscape domestication and niche construction, however, occurred long before ADEs began to be formed at Teotonio.

### Trash midden or living space?

Finally, we reflect on what the archaeobotanical data from Teotonio can tell us in terms of the context of initial ADE construction at the site.

The first clue comes from the macrobotanical data from Units 1 and 2, where high charcoal fragmentation and burnt wood frequencies of between 60–80% can be most parsimoniously associated with a domestic context, as opposed to a waste disposal area. As well as abundant wood charcoal, it interesting to note the unusually small frequencies (< 2%) of palm macroremains–not only because palms consistently make up the majority of seed and fruit remains preserved in Amazonian archaeological sites [82,83], but because it is at odds with the dominance (> 50%) of palm phytoliths in the corresponding microbotanical record. Such disparity is reconcilable if this area was originally a domestic space since, in this scenario, the palm phytoliths could have come from decomposed housing material (e.g. palm frond thatch rooves) rather than seeds discarded after fruit consumption (which constitute the macrobotanical evidence). Furthermore, the decline in phytolith recovery in the bottom 30 cm of the Massangana ADE Unit 1, and the near absence of phytoliths at 50–60 cm, might be explained by the maintenance of a `clean`living space either within, or in the immediate vicinity of, such a space. Palm phytolith frequencies in this unit are similar to those reported in another Massangana context analyzed by McMichael et al. [84] in a different area of Teotonio site (40–75%).

In Unit 5, for which macrobotanical samples unfortunately do not exist, much lower palm phytolith abundances (22–40%) and a higher input of arboreal phytoliths point towards the presence of a different activity area just 5 metres away from Unit 1. We speculated before, based on the presence of manioc and squash phytoliths and the deeper stratigraphy of this unit, that the ADEs here may have accumulated in a trash midden context, but further excavations are needed to confirm this.

The possibility of Massangana ADE accumulation in a domestic context is intriguing given ethnographic accounts of “proto-ADE” formation in house gardens [85,86], and in close proximity to dwelling spaces [87], through the piling up and burning of domestic and out-house debris. Current geochemical and micromorphological analyses, as well as future fieldwork at Teotonio should be able to shed more light on this issue.

## Conclusion

The *Cachoeira de Teotonio* in the Upper Madeira river was an important cultural hub from the early Holocene until the present day. Aside from being an unrivalled fishing spot, it was also the second waterfall in this stretch of river that could only be passed overland–a factor which would have made it an important trade and communication centre from the earliest of times.

The evidence we have presented for manioc and squash cultivation associated with ADE contexts dating to between 6,500–5,500 cal. BP, is among earliest recorded presence of these domesticates in the Amazon lowlands, alongside evidence from the Llanos de Mojos [65] and the Abeja and Peña Roja sites of the Colombian Amazon [41]. The presence of *Phaseolus* bean starch from lithic tools within the same contexts also complements evidence from the coastal Guianas [88] that this crop is of considerable antiquity in the South American lowlands.

We have argued that the adoption of exotic crops by the inhabitants of Teotonio was made more viable during the Massangana phase, when the appearance of nutrient-rich soils accumulated through increased sedentism–including in domestic and home garden contexts–created more amenable environments for these plants to adapt and grow. We posit that the uptake of exotic, more demanding crops during Massangana phase occupations can be interpreted as an increased investment in cultivated resources during this time. However, the palm and Brazil nut evidence are strong indicators that crop cultivation was situated within a mixed economy which also depended upon the exploitation and, likely, concentration, of resources on the wild to semi-domesticated spectrum–as has been posited in other tropical forest regions of the world (*sensu* [89])

We have also shown, however, that this mixed subsistence strategy was already being practiced by Teotonio´s early Holocene inhabitants during the Girau phase. As well as exploiting fruits and nuts, our evidence for tuber and root exploitation, which likely included leren and manioc cultivation, implies that societies in the upper Madeira were investing in low-level food production (*sensu* [90]) before 6,000 cal. BP, much earlier than previously thought. In fact, our preliminary evidence exhibits some similarities with the cultural sequence for the Colombian Amazon–wherein wild palm harvesting and tuber cultivation created anthropogenic forest patches as far back as 10,000–8,000 years ago [50,74].

So, if the Girau-phase inhabitants were not solely engaging in hunting and gathering, can we still consider Massangana phase occupations typical of ‘incipient agriculturalists’, who simply supplemented hunted and gathered resources with cultivated ones (*sensu* [6])? Such economic divisions are problematic, not only because of the great preceding time-depth of plant domestication and cultivation [91], but also because the relative input of cultivated vs. gathered resources is impossible to quantify for the period. Instead, we prefer to apply Killion’s (2013, p. 579 [89]) looser definition of the “hunter-fisher-gardener” society, corresponding to tropical mobile or semisedentary groups that were attracted to areas of aquatic resource abundance (the Teotonio waterfall), and which engaged in “low-impact” (rather than “low-level”) food production.

Finally, our data contribute to a growing body of evidence that places the upper Madeira and southwest Amazonia at the forefront of early cultural developments–not just in lowland South America, but within the Americas as a whole [1,92–94]
